# Is Research Experience Detrimental to a Clinical Pharmacist’s Career?

**DOI:** 10.3390/pharmacy6040105

**Published:** 2018-09-27

**Authors:** Roxanna S. D. Mohammed, Eugene Y. H. Yeung

**Affiliations:** Faculty of Medicine, The Ottawa Hospital General Campus, The University of Ottawa, Ottawa, ON K1H 8L6, Canada; rmoha018@uottawa.ca

**Keywords:** clinical pharmacist, pharmacy research, interprofessional, multi-disciplinary, physician, prejudice, publication, collaboration, education, residency

## Abstract

A recent article in the Canadian Journal of Hospital Pharmacy discussed pharmacists’ perception of clinical research. The article illustrated that pharmacists lack the time, resources, and skills to conduct research. In the current paper, two ex-pharmacists, who now work as physicians, commented on the prejudice towards pharmacy researchers. Pharmacy researchers face obstacles such as being mislabeled as “non-clinical” and lacking opportunities to be involved in high-impact publications. The current paper discussed ways to improve pharmacy research, including collaboration with well-established researchers, putting less emphasis on the “clinical” pharmacist title, and changing the pharmacy culture.

We read with interest the recent study by Lee and colleagues on pharmacists’ perception of clinical research, published in the Canadian Journal of Hospital Pharmacy [1]. We are surprised that prejudice is not listed as a barrier to conducting research. Working as pharmacists in the past and physicians now, we notice that many pharmacy colleagues like to label whether a pharmacist is clinical or not. Ironically, “clinical” research experience is not always considered to be “clinical” in pharmacy [2]. Similarly, community pharmacists, who often directly interact with patients on over-the-counter drug selections and problem-solve drug-related problems, are “non-clinical” in some pharmacists’ views [3]. Studies in Australia and the United Kingdom showed that less than half of the surveyed community pharmacists have past research experience [4,5]—this can give a false impression that pharmacists do no research. Some community pharmacy owners conduct business research, such as surveys and service evaluation [6]; unfortunately, this type of research may not be perceived as clinically relevant.

We notice that having ample research experience can give other pharmacists an impression of lack of “clinical” experience. In 2008, the American College of Clinical Pharmacy released a white paper, which mentioned that pharmacists functioning primarily as researchers would require a different set of competencies [7]. Prejudice towards researchers can be detrimental to a pharmacist’s career, especially when one applies for a pharmacy residency, post-graduate Pharm D, and post-doctoral fellowship programs. We hear Lee and colleagues’ suggestion on protected research time for pharmacists. However, we are concerned whether a pharmacy interviewer could negatively assume that these pharmacists are “non-clinical” candidates, who lack clinical competencies.

Prejudice towards researchers is possibly implied in Lee and colleagues’ study findings. A quarter of the hospital pharmacists surveyed listed the lack of management support as a barrier to conducting research; about 40% of them are looking for opportunities to join existing research teams and mentorship programs. This lack of support and opportunities could be due to their seniors not believing that research can benefit pharmacists. Although Lee’s study collected open answers, it did not publish a single excerpt from the respondents’ open answers, and mentions whether prejudice is perceived among the respondents. We wonder what response would have been given if the survey specifically asked about prejudice.

At times, we wonder whether pharmacists are too obsessed with the title “clinical” pharmacists and see research as a roadblock to their clinical careers. The British Columbia Pharmacy Association president commented that the title “clinical” pharmacist is very loosely defined [8]. A semi-structured interview suggested that pharmacists confer trust based on one’s title, degree, and status, whereas physicians confer trust based on one’s competency and performance [9]. Contrary to pharmacists, our medical colleagues tend to refer to all pharmacy colleagues as simply pharmacists, rather than adding the word “clinical” in front of their titles. Comparatively speaking, we have seldom heard of physicians self-labelling themselves as “clinical physicians”. A search of the term “clinical pharmacist” or “clinical pharmacists” on PubMed generated 2631 results, compared to 312 results for the terms “clinical physician” or “clinical physicians” (as of 21 June 2018). Adding the word “clinical” in front of our physician titles would not give us pride. It is known that all licensed physicians must be clinically competent to perform their duties, regardless of specialties. Self-labeling as “clinical” pharmacists could imply that other pharmacy colleagues are clinically incompetent. Perhaps it is time to show more respect and confidence towards different types of pharmacist, regardless of their job settings.

Lee and colleagues’ paper showed that the most common studies conducted by pharmacists are chart reviews and surveys. In an international setting, these studies may be considered as audits, which tend to have lower impacts than research [10,11]. These studies are not comparable to randomized controlled trials and systematic reviews, which have higher levels of evidence [12]. We would like to encourage pharmacists to be more involved in higher impact studies and publications.

Although 90% of the hospital pharmacists surveyed expressed interest in conducting research, the survey was conducted on a high proportion of respondents who had prior research experience, which does not represent the true general population of hospital pharmacists [13]. Those experiencing prejudice towards pharmacy researchers might have been in the large proportion of non-respondents, who are being completely put off by research. As illustrated in the study by Lee and colleagues, pharmacists lack the time, resources, and skills to conduct research. But these are common problems among many other healthcare professionals—how do they conduct research then? Were they simply born with more time and silver spoons in the mouths? It has been suggested that pharmacists’ personality traits, such as a lack of confidence and fear of new responsibility, are the ultimate barriers to pharmacy practice change [14]. Lee and colleagues did not show sufficient evidence that pharmacy researchers have more time and resources compared to non-researchers. However, the study showed that 81% of the pharmacists never apply for a research grant, but 61% complain of a lack of resources to conduct high-level studies. It appears that most of the participants unrealistically expect to receive charity funding without exerting any effort. 

What would PhD researchers do if they lacked the funding and experience to conduct a well-meaning research project? They would collaborate with experienced researchers in other facilities to gain the much-needed experience and strengthen their portfolios, before applying for additional funding to conduct independent research. Pharmacists should consider the same approach if they identify a lack of resources and skills as the main barriers to their research development. Primary care leaders are keen to improve the academic portfolios of general practitioners [15]—this is an excellent opportunity for pharmacist-physician collaboration in research. It takes time, effort, and people to change the pharmacy culture. We look forward to seeing more encouragement for pharmacist involvement in research.
